# Concerns, Mental Health, and Quality of Life in Living Kidney Donation–Parent Donor Candidates Worry Less about Themselves

**DOI:** 10.3389/fpsyg.2017.00564

**Published:** 2017-04-11

**Authors:** M. Ángeles Pérez-San-Gregorio, Agustín Martín-Rodríguez, Asunción Luque-Budia, Rupert Conrad

**Affiliations:** ^1^Department of Personality, Assessment, and Psychological Treatment, University of SevilleSeville, Spain; ^2^Liaison Mental Health Services, University Hospital Virgen del RocíoSeville, Spain; ^3^Department of Psychosomatic Medicine and Psychotherapy, University of BonnBonn, Germany

**Keywords:** living kidney donor candidates, concerns, anxiety, depression, quality of life

## Abstract

Even though the majority of living kidney donor candidates appear in good mental health and show few concerns little is known concerning the influence of the type of donor-recipient relationship on donor candidates’ specific concerns with regard to kidney donation. 136 donor candidates at Virgen del Rocío University Hospital of Seville filled in the *Scale of Concerns Regarding Living Kidney Donation* of whom 105 donor candidates and their corresponding recipients (105 patients with End-Stage Renal Disease) were further evaluated with regard to mental health (*Hospital Anxiety and Depression Scale*, *Beck Depression Inventory-II*) and quality of life (*SF-36 Health Survey*). As hypothesized recipients scored higher on depression and lower on quality of life. Donor candidates intending to donate to their children were significantly less concerned about risks of donation for themselves compared to donor candidates donating to siblings. Our findings highlight the importance of the type of donor-recipient relationship to understand specific concerns of donor candidates and optimize psychosocial assessment and support. From an evolutionary perspective parents lack of concern about their own well-being can be seen as an altruistic behavior to increase children’s fitness at the (potential) expense of their own fitness.

## Introduction

Living kidney donation is a very complex psychological experience for donor candidates as well as recipients. On the one hand, donor candidates may feel under emotional pressure because without their donation recipients’ quality of life and health may further deteriorate (Burroughs et al., 2003; Waterman et al., 2004), on the other, recipients may feel guilty for endangering donors’ health (Schweitzer et al., 2003; Hanson et al., 2015). This makes the pre-transplant stage a time of hope, vulnerability, worry and conflict (Agerskov et al., 2015). The psychosocial assessment of donors and recipients is an opportunity to speak about these concerns, identify potential ambivalences and potentially pave the path toward a solution of these conflicts. Major concerns of donor candidates refer to their own health during the donation process with key issues such as surgery, recovery and long-term risks as well as the recipients’ health with key issues such as short and long-term organ rejection. The more preoccupied donor candidates are, the more concerns about their own as well as recipients’ health one would expect. This was confirmed in a previous study on female donor candidates (Pérez-San-Gregorio et al., 2015). However, to date no study investigated the influence of the type of donor-recipient relationship on extent and content of donor candidates’ concerns. Even though mental health (Lopes et al., 2011) and quality of life (O’Driscoll et al., 2008; Lopes et al., 2013) of donor candidates is usually significantly better in comparison to recipients as well as in comparison to population samples (Lumsdaine et al., 2005; O’Driscoll et al., 2008; Pérez-San-Gregorio et al., 2015), the emotional stress due to unsolved inner conflicts may increase psychic vulnerability and predispose to the development of mental disorder such as anxiety or depression. Thus, a previous study (Conrad et al., 2016) shed light on inner conflicts of highly reward dependent donor candidates who are particularly sensitive to both reward and punishment in social settings. These candidates are predisposed to strongly feel the moral obligation to help. The moral obligation on the one hand and the fear of negative consequences to one’s own health on the other hand are conflicting attitudes. This cognitive dissonance leads to mental distress and may be solved by the denial of risks of donation. A large study on the well-being of donors identified a feeling of moral obligation to donate as a risk factor for the development of depression after donation (Jowsey et al., 2014).

Given the importance of this subject in clinical practice we aimed at investigating whether donor candidates specific concerns in relation to kidney donation differ depending on type of donor-recipient relationship. Furthermore, as to date no study investigated mental health and quality of life in donor and recipient candidates in Spain, we took a closer look at these aspects. Previous studies from other European countries and the US on recipients’ quality of life and mental health demonstrated significant pre- to post-transplant improvements (Dew et al., 1997). In specific areas such as social functioning and mental health the post-transplant quality of life was comparable to the healthy population whereas in other areas such as physical functioning it did not equal healthy cohorts (Neipp et al., 2006). Living kidney donors have been found to have high health-related quality of life before transplantation, which even surpasses quality of life in the general population (Wirken et al., 2015). Shortly after donation, donors’ quality of life decreases with major changes in physical functioning and moderate changes in psychological functioning. In the course of 3–12 months in the vast majority of donors a normal level of health-related quality of life is restored and remains stable (Wirken et al., 2015). In a recent study on long-term outcome in 2455 kidney donors only 1% of donors reported that donation affected their health very negatively (Gross et al., 2013). Across studies impaired mental health before donation has been identified as the most important risk factor for impaired long-term health-related quality of life (Wirken et al., 2015).

We hypothesized that donor candidates compared to recipients would show significantly lower scores on depression and higher scores on quality of life. Furthermore, we hypothesized that parent donor candidates would show more concerns about recipients and less concerns about themselves compared to donating siblings.

## Materials and Methods

### Participants

A sample of 136 consecutive donor candidates was investigated by the *Scale of Concerns Regarding Living Kidney Donation* (Pérez-San-Gregorio et al., 2015). 105 of these donor candidates and their corresponding recipients (105 patients with End-Stage Renal Disease, ESRD) were assessed by the *Hospital Anxiety and Depression Scale* (HADS; Zigmond and Snaith, 1983), the *Beck Depression Inventory-II* (BDI-II; Beck et al., 1996), and the *SF-36 Health Survey* (SF-36; Alonso et al., 1995).

The group of donor candidates was made up of 43 men and 93 women with a mean age of 47.68 years (*SD* = 10.42 years). The relationship with their recipient was as follows: parent (39%), partner (33.8%), sibling (20.6%), other (6.6%). The group of recipient candidates was comprised of 105 patients (66 men and 39 women) with a mean age of 43.23 years (*SD* = 2.82 years). The mean time of duration of illness was 14.74 years (*SD* = 12.72 years). The causes of kidney failure included chronic glomerulonephritis (49.5%), renal cystic diseases and renal dysplasia (17.1%), other (14.3%), tubulointerstitial nephropathy (10.5%), and chronic renal failure of unknown origin (8.6%).

The mental health and quality of life of the donor candidates and their recipients were compared with population samples from previous studies representative of the Spanish adult population, which consisted of 182 (HADS; Terol et al., 2007), 569 (BDI-II; Sanz et al., 2014), and 8778 (SF-36; Alonso et al., 1998) participants.

### Instruments

*Hospital Anxiety and Depression Scale* (Zigmond and Snaith, 1983). This instrument consists of 14 items, seven on depression and seven on anxiety, in which patients indicate how they felt during the past week, selecting one of four response options. The test provides two scores, one for anxiety and the other for depression. Each of these scores can vary from 0 to 21. Higher scores indicate more anxious-depressive symptomatology. We used the Spanish version developed by Caro and Ibáñez (1992). In our sample Cronbach’s alpha was 0.76 (donor candidates) and 0.84 (recipient candidates) for the anxiety subscale and 0.78 (donor candidates) and 0.80 (recipient candidates) for the depression subscale.

*Beck Depression Inventory-II* (Beck et al., 1996). This questionnaire consists of 21 items which evaluate the severity of depressive symptomatology in the past 2 weeks. The items are rated on a four-point scale ranging from no symptoms to very severe symptoms, except for items 16 and 18 which are rated on a seven-point rating scale. The total score can vary from 0 to 63. Higher scores show more severe depressive symptomatology. We used the Spanish version developed by Beck et al. (2011). In our sample Cronbach’s alpha was 0.88 (donor candidates) and 0.93 (recipient candidates).

SF-36 (Alonso et al., 1995). This instrument is made up of 36 items, each with several response choices, which together provide a health status profile. The test explores eight dimensions: physical functioning, role limitations due to physical problems, bodily pain, general health perceptions, vitality, social functioning, role limitations due to emotional problems, and general mental health. In each dimension, the score ranges from 0 (worse health status) to 100 (better health status). In our sample Cronbach’s alpha across various dimensions varied from 0.46 to 0.84 with a mean of 0.73 for donor candidates, and from 0.62 to 0.94 with a mean of 0.83 for recipient candidates.

*Scale of Concerns Regarding Living Kidney Donation* (Pérez-San-Gregorio et al., 2015). This scale is made up of 14 items, four items evaluating concerns of the donor candidates relating to the recipients (Factor I), and 10 items evaluating concerns of the donor candidates relating to themselves (Factor II). The items are rated on a four-point scale ranging from none at all to a lot. In a further step of analysis, the mean score on both factors, which varied from 1 (no concern) to 4 (maximum concern) was taken into account. In our sample Cronbach’s alpha was 0.83 for Factor I and 0.84 for Factor II.

### Procedure

After receiving Institutional Review Board approval for the study, the data were collected from January 2013 to September 2016 at Virgen del Rocío University Hospital of Seville. First, the Nephrology Clinical Management Unit screened the candidate donor and recipient pairs who were medically suitable for living kidney transplantation and who desired to undergo this procedure. At this stage there was no psychosocial evaluation or mental health screening. Afterward all of the 140 donor-recipient pairs were referred to the Mental Health Clinical Management Unit for further psychosocial assessment. Inclusion criteria for our study were: (a) over 18 years of age, (b) informed consent, (c) no difficulties in understanding the evaluation instruments, and (d) no severe or disabling psychopathological condition. Four donor candidates (two did not wish to participate and two did not understand Spanish) and 35 recipient candidates (28 were minors, five did not wish to participate and two had slight intellectual disabilities) had to be excluded. Thus, 136 donor candidates and 105 donor-recipient pairs could be included in the study.

### Statistical Analysis

The data were analyzed with the SPSS 22 statistics program. We used the paired *t*-test to analyze differences in mental health and quality of life between the two paired groups of donor and recipient candidates and independent *t*-test for the comparison with population samples. To analyze differences in living kidney donor candidates concerns due to type of donor-recipient relationship (parent, sibling, partner, and other) we used the Kruskal–Wallis *H* test followed by the Dunn-test adjusted for multiple comparisons. Furthermore, Cohen’s *d* was calculated as a measure of effect size for all comparisons in the study.

## Results

### Mental Health

**FIGURE 1:**
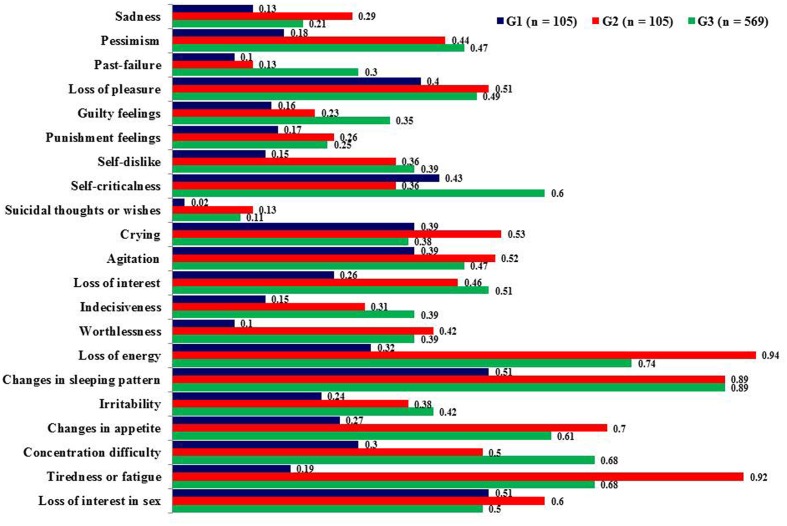
**Mean scores on *Beck Depression Inventory-II* (BDI-II) items**. Higher scores show more depressive symptomatology, G1 = Living kidney donor candidates, G2 = Living donor kidney transplant recipient candidates, G3 = General Spanish population.

There was no difference between donors and recipients regarding self-reported anxiety. Potential recipients scored significantly higher on depression compared to donor candidates as measured by BDI-II (**Table 1**). Regarding specific depressive symptomatology they showed higher scores on worthlessness (*p* < 0.001, *d* = -0.54), loss of energy (*p* < 0.001, *d* = -0.91), changes in appetite (*p* < 0.001, *d* = -0.65), and tiredness or fatigue (*p* < 0.001, *d* = -1.12) (**Figure 1**).

**Table 1:** Differences between donor and recipient candidates in mental health.

	G1	G2	*t*_(104)_	Cohen’s *d*
	(*n* = 105)	(*n* = 105)	(*p*)	
	(*SD*)	(*SD*)		
**HADS**				
Total anxiety	4.69	5.10	-0.85	-0.10
	(3.58)	(4.36)	(0.397)	N
Total depression	2.10	3.40	-3.25	-0.42

	(2.64)	(3.53)	(0.002)	S


**BDI-II**				
Total depression	5.39 (6.30)	9.90 (9.68)	-4.57 (<0.001)	-0.55 M

*G1 = Living kidney donor candidates, G2 = Living donor kidney transplant recipient candidates, N = Null effect size, S = Small effect size, M = Medium effect size. ^1^Higher scores show more anxious-depressive symptomatology*.

In comparison to the population samples (*n* = 182 for HADS; *n* = 569 for BDI-II) donor candidates showed less anxiety (*p* < 0.001, *d* = -0.90) and depression (HADS: *p* < 0.001, *d* = -0.79; BDI-II: *p* < 0.001, *d* = -0.60). The most relevant differences in depressive symptomatology were as follows: pessimism (*p* < 0.001, *d* = -0.52), worthlessness (*p* < 0.001, *d* = -0.51), loss of energy (*p* < 0.001, *d* = -0.68), concentration difficulty (*p* < 0.001, *d* = -0.58), and tiredness or fatigue (*p* < 0.001, *d* = -0.79). Recipient candidates showed less anxious symptomatology (*p* < 0.001, *d* = -0.72).

### Quality of Life

**FIGURE 2:**
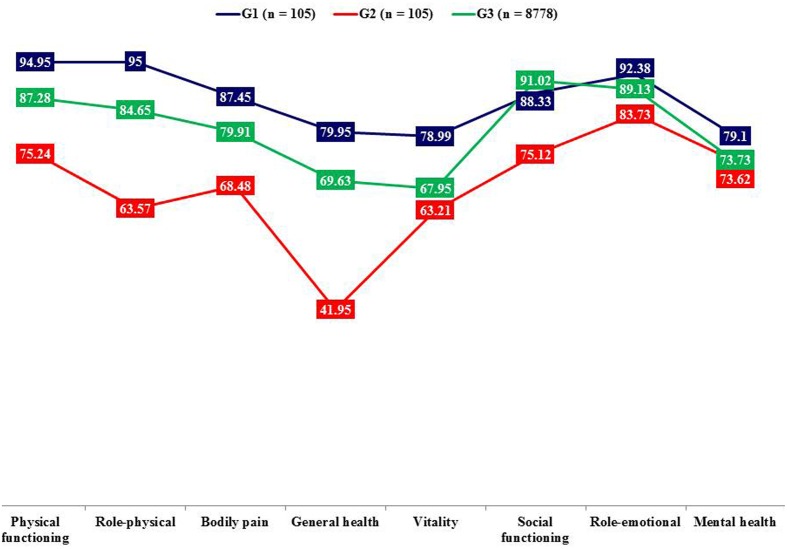
**Mean scores on SF-36 dimensions**. Lower scores show worse quality of life, G1 = Living kidney donor candidates, G2 = Living donor kidney transplant recipient candidates, G3 = General Spanish population (means adjusted to the age of the participants in this study).

Donor candidates compared to recipients showed higher quality of life on the following dimensions: physical functioning, role-physical, bodily pain, general health, vitality, and social functioning (**Table 2** and **Figure 2**).

**Table 2:** Differences between donor and recipient candidates in quality of life (SF-36).

	G1	G2	*t*_(104)_	*p*	Cohen’s *d*
	(*n* = 105)	(*n* = 105)	*t*_(104)_	*p*	Cohen’s *d*
	G1	G2	*t*_(104)_	*p*	Cohen’s *d*
	Mean^1^	(*n* = 105)			
Physical functioning	94.95 (8.97)	75.24 (24.18)	7.84	<0.001	1.08 L
Role-physical	95.00 (10.91)	63.57 (30.22)	10.66	<0.001	1.38 L
Bodily pain	87.45 (15.29)	68.48 (28.94)	5.94	<0.001	0.82 L
General health	79.95 (14.52)	41.95 (18.51)	16.62	<0.001	2.28 L
Vitality	78.99 (15.26)	63.21 (22.37)	6.79	<0.001	0.82 L
Social functioning	88.33 (18.85)	75.12 (23.80)	4.96	<0.001	0.61 M
Role-emotional	92.38 (13.23)	83.73 (23.69)	3.59	0.001	0.45 S
Mental health	79.10 (16.70)	73.62 (20.41)	2.43	0.017	0.29 S

*G1 = Living kidney donor candidates, G2 = Living donor kidney transplant recipient candidates, S = Small effect size, M = Medium effect size, L = Large effect size. ^1^Lower scores show worse quality of life*.

In comparison to the population sample (*n* = 8778) donor candidates showed higher quality of life on general health (*p* < 0.001, *d* = 0.56) and vitality (*p* < 0.001, *d* = 0.59) dimensions. On the contrary, the recipient candidates displayed poorer quality of life on physical functioning (*p* < 0.001, *d* = -0.53), role-physical (*p* < 0.001, *d* = -0.66), general health (*p* < 0.001, *d* = -1.38), and social functioning (*p* < 0.001, *d* = -0.74).

### Concerns Regarding Living Kidney Donation

On the basis of donor-recipient relationship donor candidates were divided into four groups. Between these groups there were no differences with regard to gender, age, depression, and anxiety (HADS). Analysis of donor candidates’ concerns displayed no differences with regard to concerns related to recipients’ well-being. In accordance with our hypothesis parent donor candidates showed less concerns relating to themselves (Factor II) compared to donating siblings (**Table 3**). Analysis of the different items subsumed under Factor II showed that parent donor candidates worried less about having kidney complications for the rest of their lives, a member of the family not accepting their decision, the donation having repercussions on their daily lives, or that having to live with only one kidney could be detrimental to their job or their health (**Table 4**).

**Table 3:** Concerns of the living kidney donor candidates based on their relationship with the recipient.

	Parent (*n* = 53) Mean^1^ (*SD*) a	Sibling (*n* = 28) Mean (*SD*) b	Partner (*n* = 46) Mean (*SD*) c	Other (*n* = 9) Mean (*SD*) d	χ^2^_(3,136)_ (*p*)	Dunn’s test (*p*) [Cohen’s *d*]
						
						a–b	a–c	a–d	b–c	b–d	c–d
Concerns of the donor candidates relating to the recipients	2.86 (0.94)	2.96 (0.76)	2.89 (0.81)	3.33 (0.60)	2.02 (0.567)	–	–	–	–	–	–
Concerns of the donor candidates relating to themselves	1.59 (0.46)	2.01 (0.76)	1.72 (0.51)	1.76 (0.27)	8.82 (0.032)	-25.54 (0.032) [-0.67] M	-12.11 (0.758) [-0.27] S	–22.75 (0.650) [-0.45] S	13.43 (0.924) [0.45] S	2.79 (1.000) [0.44] S	-10.64 (1.000) [-0.10] N

*N = Null effect size, S = Small effect size, M = Medium effect size. ^1^Higher scores show more concern*.

**Table 4:** Concerns of the donor candidates relating to themselves: comparison between parent and sibling donor candidates.

	Parent (*n* = 53) Mean^1^ (*SD*)	Sibling (*n* = 28) Mean (*SD*)	Z (*p*)	Cohen’s *d*
Having any complications during the operation	1.98 (1.01)	2.32 (1.12)	-1.32 (0.186)	-0.32 S
Having any postoperative complications	1.91 (0.88)	2.32 (0.98)	-1.92 (0.055)	-0.44 S
Having something that would prevent me from donating	2.81 (1.27)	2.79 (1.26)	-0.04 (0.966)	0.02 N
That my family members could not take care of me or assist me during my stay at the hospital	1.51 (0.97)	1.50 (0.96)	-0.10 (0.918)	0.01 N
Having any kidney complications throughout my life	1.57 (0.89)	2.18 (1.02)	-2.95 (0.003)	-0.64 M
That a family member would not accept my decision	1.06 (0.30)	1.82 (1.31)	-3.61 (<0.001)	-0.80 L
That the donation could affect my daily life	1.32 (0.64)	2.11 (1.17)	-3.47 (0.001)	-0.84 L
That living with one kidney could hurt me at my job	1.30 (0.72)	1.89 (1.13)	-2.73 (0.006)	-0.62 M
That living with one kidney could hurt my relationships with others	1.15 (0.63)	1.25 (0.58)	-1.61 (0.107)	-0.16 N
That living with one kidney could hurt my health	1.28 (0.57)	1.96 (1.00)	-3.55 (<0.001)	-0.83 L

*N = Null effect size, S = Small effect size, M = Medium effect size, L = Large effect size. ^1^Higher scores show more concern*.

## Discussion

Our cross-sectional study aimed at comparing for the first time mental health and quality of life between Spanish living kidney donor candidates and their paired recipients. Donor candidates displayed less depressive symptomatology such as worthlessness, loss of energy, changes in appetite, and fatigue as well as better quality of life than recipients, which is in line with previous studies from other countries (O’Driscoll et al., 2008; Lopes et al., 2011, 2013). Particularly symptoms such as loss of energy, tiredness and fatigue may reflect somatic symptoms due to dialysis and/or renal dysfunction in ESRD (Pérez-San-Gregorio et al., 2007). The overlap between symptoms typical for severe organic malfunction as well as depression may complicate the diagnosis of depression in kidney recipient candidates and highlights the necessity of an expert psychosocial assessment as essential part of the medical procedure. This symptomatology goes hand in hand with lower quality of life on different dimensions such as physical and social functioning. On the other hand there were no differences between donor and recipient candidates with regard to anxiety and they showed even less anxiety compared to a population sample, which is in keeping with a previous study (Lumsdaine et al., 2005). One might argue that the imminent perspective of a life without dialysis increases psychological well-being and renders other concerns irrelevant.

Furthermore, donor candidates compared to the population sample showed less anxious-depressive symptomatology (pessimism, worthlessness, loss of energy, concentration difficulty, and tiredness or fatigue) and more quality of life (general health and vitality). These results are congruent with previous studies which have consistently come to the conclusion that donor candidates’ self-reported mental health as well as quality of life are excellent (Lumsdaine et al., 2005; O’Driscoll et al., 2008; Pérez-San-Gregorio et al., 2015; Wirken et al., 2015). The exchange gift theory argues that for donors and donor candidates the willingness to living kidney donation is intrinsically rewarding, because the return for most donors is contained in the act of giving itself and the associated personal transformation (Gill and Lowes, 2008). In this way the willingness to living kidney donation may increase self-esteem (Tanriverdi et al., 2004; Garcia et al., 2013; Joshi et al., 2013; Hildebrand et al., 2014) and generate psychological benefits like pride and increased self-awareness. Moreover the willingness to donate may inspire gratitude as well as respect of significant others (Rodrigue et al., 2011). This may contribute to an increase in psychological well-being and quality of life (Feltrin et al., 2008). Besides donor candidates are a highly selective group which were included after having undergone strict medical testing. Therefore it is ensured that candidates do not suffer from chronic diseases such as diabetes, cancer, kidney or heart disease, which makes a high level of physical and social functioning likely (Maglakelidze et al., 2011). However, this self-assessment also could be the result of a defense or coping strategy centered around the denial of donation risks to their own health (Pradel et al., 2003; Lumsdaine et al., 2005; Feltrin et al., 2008; O’Driscoll et al., 2008; Kroencke et al., 2012). Finally, donor candidates are well aware of the importance of adequate physical as well as mental health to be assessed as fit for donation. Therefore, self-report questionnaires on mental health and quality of life may be answered in the socially desired way (Erim et al., 2015).

Analysis of donor candidates’ concerns found significant differences between parents and siblings. Thus, parents worried less about the negative repercussions of nephrectomy such as kidney complications, problems in daily life, work life and general health problems. Along this line, other studies indicated that parents perceive their donation as a natural gift to their children, while for siblings it could be a moral obligation to meet family expectations (Crombie and Franklin, 2006; Tong et al., 2012). An interesting investigation by Zeiler et al. (2010) analyzed parents’ narratives on living kidney donation and highlighted the presence of a parenthood moral imperative of always putting one’s child’s needs before one’s own. This imperative may seriously affect autonomous decision making, if its internalization is not the result of parents’ autonomous choice and if the imperative makes parents unable to decline a donation to their children (Zeiler et al., 2010).

From an evolutionary perspective this parental behavior roots in a biologically founded form of altruism (Preston, 2013). Altruistic responding becomes apparent when parents help their children at actual or potential cost to themselves. The instincts underlying this behavioral pattern aim at maximizing fitness and the chance of reproductive success of one’s offspring (Preston, 2013). From a psychological perspective altruism may imply parents’ denial of personal risks of nephrectomy in order to be able to alleviate their children’s suffering. However, this does not necessarily mean that parental donors are more resilient regarding the impact of negative consequences of donation on mental health. Therefore, in the medical assessment altruism must be balanced with consideration of the risks and benefits, to guarantee the best course of action for donor and recipient (Freeman, 2012).

There is growing evidence that a comprehensive psychosocial assessment of living kidney donors and recipients is necessary to guarantee the best possible outcome (Sajjad et al., 2007; Conrad et al., 2016). From a clinical perspective our findings are significant, because they may help to optimize the quality of psychosocial assessment in donor candidates. In parental donor candidates it might be important to question the above mentioned parenthood moral imperative, which may seriously hamper autonomous decision-making. Moreover, it can be necessary to counteract parents’ tendency to deny negative repercussions for themselves, because the realistic anticipation of possible consequences in the context of psychosocial assessment/counseling is an important prevention strategy against the development of post donation mental health problems (Schweitzer et al., 2003; Waterman et al., 2004; de Groot et al., 2012). In donating siblings it may be important to identify an unsolved conflict between a strong feeling of moral obligation to donate on the one hand and strong concerns regarding personal health on the other, which may deeply affect deliberate decision-making and predispose to the development of depression after donation (Dew et al., 2013; Jowsey et al., 2014). Particularly in individuals feeling highly socially dependent the psychosocial assessment may prove challenging because candidates present a socially desired conflict-free façade (Conrad et al., 2016).

Limitations of the study that need to be addressed include cross-sectional study design that implies there is no post donation assessment to further help demonstrate the importance of the issue. Furthermore, it would have been of interest to compare our results with a control group of healthy individuals (Maglakelidze et al., 2011). Besides, we did not assess the personality of donor candidates, even though personality can have an important impact on motivation and concerns in addition to the type of donor-recipient relationship (Conrad et al., 2016). Finally, our study focused on concerns of donor candidates, however, in future studies it would be important to also analyze recipients’ concerns as well as the mutual relationship between concerns in donor-recipient pairs. A special strength of the study is the large sample size and the low drop out rate in donor candidates, which enhances external validity of findings.

## Ethics Statement

Ethics Committee of the Virgen del Rocío University Hospital of Seville. All subjects gave written informed consent in accordance with the Declaration of Helsinki.

## Author Contributions

MÁP-S-G: Study concept and design, data analysis and interpretation, drafting of manuscript, manuscript revisions, and drafting figures. AM-R: Study concept and design, data analysis and interpretation, manuscript revisions. AL-B: Institutional support, data collection, critical revision of article. RC: Data analysis and interpretation, drafting of manuscript, critical revision of article. MÁP-S-G, AM-R, AL-B, and RC: Final approval of version to be published.

## Conflict of Interest Statement

The authors declare that the research was conducted in the absence of any commercial or financial relationships that could be construed as a potential conflict of interest. The reviewer (SC) and the handling Editor declared their shared affiliation, and the handling Editor states that the process nevertheless met the standards of a fair and objective review.

## References

[B1] Agerskov H. Ludvigsen M. S. Bistrup C. Pedersen B. D. (2015). Living kidney donors’ experiences while undergoing evaluation for donation: a qualitative study. *J. Clin. Nurs.* 24 2258–2267. 10.1111/jocn.12776 25753175

[B2] Alonso J. Prieto L. Antó J. M. (1995). The Spanish version of the SF-36 Health Survey (SF-36 health questionnaire): an instrument for measuring clinical results. *Med. Clin.* 104 771–776. 7783470

[B3] Alonso J. Regidor E. Barrio G. Prieto L. Rodríguez C. de la Fuente L. (1998). Population-based reference values for the Spanish version of the Health Survey SF-36. *Med. Clin.* 111 410–416. 9834913

[B4] Beck A. T. Steer R. A. Brown G. K. (1996). *BDI–II. Beck Depression Inventory Manual*, 2nd Edn San Antonio, TX: The Psychological Corporation.

[B5] Beck A. T. Steer R. A. Brown G. K. (2011). Manual BDI-II. Inventario de Depresión de Beck-II (Adaptación española: J. Sanz and C. Vázquez). Madrid: Pearson.

[B6] Burroughs T. E. Waterman A. D. Hong B. A. (2003). One organ donation, three perspectives: experiences of donors, recipients, and third parties with living kidney donation. *Prog. Transplant.* 13 142–150. 10.7182/prtr.13.2.71t8xj210l18mx25 12841522

[B7] Caro I. Ibáñez E. (1992). The hospital anxiety and depression scale. Its practical utility in health psychology. *Bol. Psicol.* 36 43–69.

[B8] Conrad R. Kleiman A. Rambau S. Wegener I. Mücke M. Dolscheid-Pommerich R. C. (2016). Psychosocial assessment of living kidney donors: what implications have temperament and character for decision-making? *Compr. Psychiatry* 67 1–8. 10.1016/j.comppsych.2016.02.007 27095327

[B9] Crombie A. K. Franklin P. M. (2006). Family issues implicit in living donation. *Mortality* 11 196–210. 10.1080/13576270600616011

[B10] de Groot I. B. Stiggelbout A. M. van der Boog P. J. Baranski A. G. Marang-van de Mheen P. J. Partner-study group. (2012). Reduced quality of life in living kidney donors: association with fatigue, societal participation and pre-donation variables. *Transpl. Int.* 25 967–975. 10.1111/j.1432-2277.2012.01524.x 22780196

[B11] Dew M. A. Di Matirni A. F. DeVito Dabbs A. J. Zuckoff A. Tan H. P. McNulty M. L. (2013). Preventive intervention for living donor psychosocial outcomes: feasibility and efficacy in a randomized controlled trial. *Am. J. Transplant.* 13 2672–2684. 10.1111/ajt.12393 23924065 PMC3837427

[B12] Dew M. A. Switzer G. E. Goycoolea J. M. Allen A. S. DiMartini A. Kormos R. L. (1997). Does transplantation produce quality of life benefits? A quantitative analysis of the literature. *Transplantation* 64 1261–1273. 10.1097/00007890-199711150-00006 9371666

[B13] Erim Y. Kahraman Y. Vitinius F. Beckmann M. Kröncke S. Witzke O. (2015). Resilience and quality of life in 161 living kidney donors before nephrectomy and in the aftermath of donation: a naturalistic single center study. *BMC Nephrol.* 16:164 10.1186/s12882-015-0160-z PMC4608317 26475323

[B14] Feltrin A. Pegoraro R. Rago C. Benciolini P. Pasquato S. Frasson P. (2008). Experience of donation and quality of life in living kidney and liver donors. *Transpl. Int.* 21 466–472. 10.1111/j.1432-2277.2007.00632.x 18225994

[B15] Freeman R. B. Jr. (2012). The limits of altruism: selecting living donors. *Virtual Mentor* 14 272–277. 10.1001/virtualmentor.2012.14.3.oped1-1203 23352017

[B16] Garcia M. F. F. M. Andrade L. G. M. Carvalho M. F. C. (2013). Living kidney donors – a prospective study of quality of life before and after kidney donation. *Clin. Transplant.* 27 9–14. 10.1111/j.1399-0012.2012.01687.x 22831164

[B17] Gill P. Lowes L. (2008). Gift exchange and organ donation: donor and recipient experiences of live related kidney transplantation. *Int. J. Nurs. Stud.* 45 1607–1617. 10.1016/j.ijnurstu.2008.03.004 18462738

[B18] Gross C. R. Messersmith E. E. Hong B. A. Jowsey S. G. Jacobs C. Gillespie B. W. (2013). Health-related quality of life in kidney donors from the last five decades: results from the RELIVE study. *Am. J. Transplant.* 13 2924–2934. 10.1111/ajt.12434 24011252 PMC4091665

[B19] Hanson C. S. Chadban S. J. Chapman J. R. Craig J. C. Wong G. Ralph A. F. (2015). The expectations and attitudes of patients with chronic kidney disease toward living kidney donor transplantation: a thematic synthesis of qualitative studies. *Transplantation* 99 540–554. 10.1097/TP.0000000000000433 25463967

[B20] Hildebrand L. Melchert T. P. Anderson R. C. (2014). Impression management during evaluation and psychological reactions post-donation of living kidney donors. *Clin. Transplant.* 28 855–861. 10.1111/ctr.12390 24888484

[B21] Joshi S. A. Almeida N. Almeida A. (2013). Assessment of the perceived quality of life of successful kidney transplant recipients and their donors pre- and post-transplantation. *Transplant. Proc.* 45 1435–1437. 10.1016/j.transproceed.2013.01.037 23726590

[B22] Jowsey S. G. Jacobs C. Gross C. R. Hong B. A. Messersmith E. E. Gillespie B. W. (2014). Emotional well-being of living kidney donors: findings from the RELIVE Study. *Am. J. Transplant.* 14 2535–2544. 10.1111/ajt.12906 25293374 PMC4205186

[B23] Kroencke S. Fischer L. Nashan B. Herich L. Schulz K. H. (2012). A prospective study on living related kidney donor’s quality of life in the first year: choosing appropriate reference data. *Clin. Transplant.* 26 418–427. 10.1111/j.1399-0012.2012.01691.x 22882697

[B24] Lopes A. Frade I. C. Teixeira L. Almeida M. Dias L. Henriques A. C. (2013). Quality of life assessment in a living donor kidney transplantation program: evaluation of recipients and donors. *Transplant. Proc.* 45 1106–1109. 10.1016/j.transproceed.2013.02.100 23622638

[B25] Lopes A. Frade I. C. Teixeira L. Oliveira C. Almeida M. Dias L. (2011). Depression and anxiety in living kidney donation: evaluation of donors and recipients. *Transplant. Proc.* 43 131–136. 10.1016/j.transproceed.2010.12.028 21335170

[B26] Lumsdaine J. A. Wray A. Power M. J. Jamieson N. V. Akyol M. Bradley J. A. (2005). Higher quality of life in living donor kidney transplantation: prospective cohort study. *Transpl. Int.* 18 975–980. 10.1111/j.1432-2277.2005.00175.x 16008749

[B27] Maglakelidze N. Pantsulaia T. Managadze L. Chkhotua A. (2011). Assessment of health-related quality of life in living kidney donors. *Transplant. Proc.* 43 373–375. 10.1016/j.transproceed.2010.12.016 21335225

[B28] Neipp M. Karavul B. Jackobs S. Meyer zu Vilsendorf A. Richter N. Becker T. (2006). Quality of life in adult transplant recipients more than 15 years after kidney transplantation. *Transplantation* 81 1640–1644. 10.1097/01.tp.0000226070.74443.fb 16794528

[B29] O’Driscoll C. T. House A. K. Holman C. D. (2008). Quality of life measurement using the Short Form-36: a preoperative study of liver and kidney transplantation and living kidney donation in Western Australia. *Transplant Nurses J.* 17 32–38.

[B30] Pérez-San-Gregorio M. A. Fernández-Jiménez E. Luque-Budia A. Martín-Rodríguez A. (2015). Anxiety and concerns in Spanish living kidney donor candidates. *Int. J. Psychiatry Med.* 50 163–177. 10.1177/0091217415605031 26340911

[B31] Pérez-San-Gregorio M. A. Martín-Rodríguez A. Díaz-Domínguez R. Pérez-Bernal J. (2007). Evolution of health-related quality of life in kidney transplanted patients. *Nefrologia* 27 619–626. 18045039

[B32] Pradel F. G. Mullins C. D. Bartlett S. T. (2003). Exploring donors’ and recipients’ attitudes about living donor kidney transplantation. *Prog. Transplant.* 13 203–210. 14558635 10.1177/152692480301300307

[B33] Preston S. D. (2013). The origins of altruism in offspring care. *Psychol. Bull.* 139 1305–1341. 10.1037/a0031755 23458432

[B34] Rodrigue J. R. Schutzer M. E. Paek M. Morrissey P. (2011). Altruistic kidney donation to a stranger: psychosocial and functional outcomes at two US transplant centers. *Transplantation* 91 772–778. 10.1097/TP.0b013e31820dd2bd 21285916

[B35] Sajjad I. Baines L. S. Salifu M. Jindal R. M. (2007). The dynamics of recipient-donor relationships in living kidney transplantation. *Am. J. Kidney Dis.* 50 834–854. 10.1053/j.ajkd.2007.07.029 17954298

[B36] Sanz J. Gutiérrez S. Gesteira C. García-Vera M. P. (2014). Criteria and norms for interpreting the Beck Depression Inventory-II (BDI-II). *Behav. Psychol.* 22 37–59. 10.1037/a0027920

[B37] Schweitzer J. Seidel-Wiesel M. Verres R. Wiesel M. (2003). Psychological consultation before living kidney donation: finding out and handling problem cases. *Transplantation* 76 1464–1470. 10.1097/01.TP.0000084320.57817.32 14657687

[B38] Tanriverdi N. Özçürümez G. Çolak T. Dürü C. Emiroğlu R. Zileli L. (2004). Quality of life and mood in renal transplantation recipients, donors, and controls: preliminary report. *Transplant. Proc.* 36 117–119. 10.1016/j.transproceed.2003.11.003 15013318

[B39] Terol M. C. López-Roig S. Rodríguez-Marín J. Martín-Aragón M. Pastor M. A. Reig M. T. (2007). Hospital anxiety and depression scale (HAD): psychometric properties in Spanish population. *Ansiedad Estrés* 13 163–176.

[B40] Tong A. Chapman J. R. Wong G. Kanellis J. McCarthy G. Craig J. C. (2012). The motivations and experiences of living kidney donors: a thematic synthesis. *Am. J. Kidney Dis.* 60 15–26. 10.1053/j.ajkd.2011.11.043 22305757

[B41] Waterman A. D. Covelli T. Caisley L. Zerega W. Schnitzler M. Adams D. (2004). Potential living kidney donors’ health education use and comfort with donation. *Prog. Transplant.* 14 233–240. 10.7182/prtr.14.3.v122u40686617121 15495783

[B42] Wirken L. van Middendorp H. Hooghof C. W. Rovers M. M. Hoitsma A. J. Hilbrands L. B. (2015). The course and predictors of health-related quality of life in living kidney donors: a systematic review and meta-analysis. *Am. J. Transplant.* 15 3041–3054. 10.1111/ajt.13453 26414703

[B43] Zeiler K. Guntram L. Lennerling A. (2010). Moral tales of parental living kidney donation: a parenthood moral imperative and its relevance for decision making. *Med. Health Care Philos.* 13 225–236. 10.1007/s11019-010-9238-3 20186572

[B44] Zigmond A. S. Snaith R. P. (1983). The hospital anxiety and depression scale. *Acta Psychiatr. Scand.* 67 361–370. 10.1111/j.1600-0447.1983.tb09716.x 6880820

